# Nephrolithiasis Greater Than 2 cm and Splenomegaly

**DOI:** 10.1089/cren.2015.29015.mls

**Published:** 2015-10-01

**Authors:** Maximiliano Lopez Silva, Horacio Sanguinetti, Jorge Aguilar, Adolfo Alvarez Alberó, Norberto Bernardo

**Affiliations:** Department of Urology, Hospital de Clínicas José de San Martín, Buenos Aires, Argentina.

## Abstract

A 67-year-old male presented with left kidney stones in renal pelvis, 15 mm length. Preoperative CT showed massive splenomegaly. Retrograde intrarenal surgery approach was decided to avoid splenic injury, achieving the absence of residual stones.

## Clinical History

A 67-year-old obese (body mass index = 29) male presented with surgical history of bilateral inguinal hernia repair and umbilicoplasty. Medical history includes myelofibrosis, dyslipidemia, and hypertension. He consulted in March 2015 with 3 months of left lumbar pain, with flank irradiation, no fever associated.

## Diagnostic Studies

Urinary X-ray showed no stones. Ultrasonography reported a 13-mm stone in the left renal pelvis not associated with urinary-tract dilation. A CT scan showed a 15-mm stone of the left renal pelvis associated with mild dilatation of the urinary tract and a massive splenomegaly. Left kidney was displaced medially by the enlargement of the spleen.

**FIG. 1.**
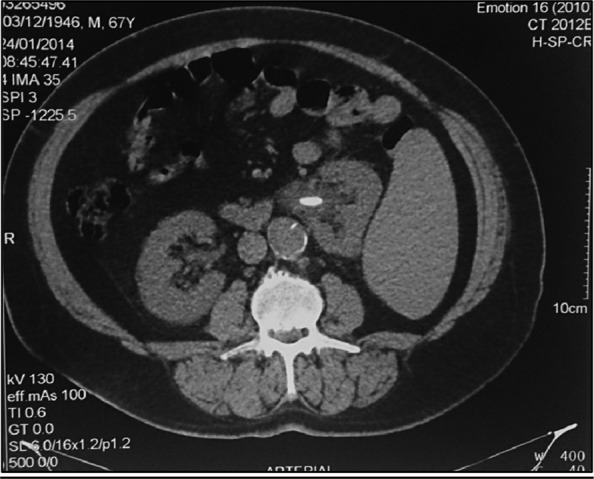
Abdominal CT that evidences splenomegaly and nephrolithiasis.

**FIG. 2.**
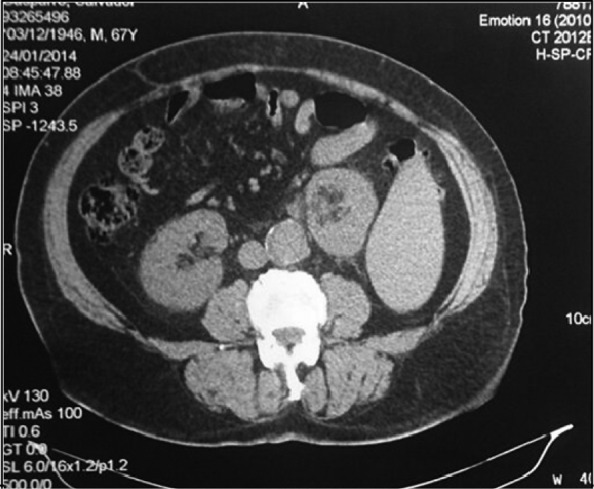
Abdominal CT that evidences splenomegaly and displacement of the left kidney.

Urine culture was negative. Laboratory tests showed normal renal function and thrombocytopenia, with mild coagulopathy (Quick test 65%). Other laboratory parameters showed no pathologic findings (Figs. 1, 2).

## Intervention

Retrograde intrarenal surgery (RIRS) was decided. The patient was placed in the lithotomy position. Cystoscopy was performed using 20F cystoscope (Karl Storz-Endoskope^®^); retrograde pyelography with fluoroscopic guidance confirmed the presence of renal pelvic and lower caliceal stones. 0.035" hydrophilic guide (Cook Medical Devices^®^) was placed.

Subsequently, semi-rigid ureteroscopy was conducted using 7.5–9F ureteroscope (Karl Storz-Endoskope), with no evidence of ureteral calculi. A second 0.035" hydrophilic guidewire (Cook Medical Devices) was placed.

Finally, RIRS was performed with a flexible ureteroscope Flex X2 (Karl Storz-Endoskope) 7.5F diameter using a 12F ureteral sheath (Cook Medical Devices). Access to the upper urinary tract was achieved without drawbacks, and lithotripsy with Holmium Laser Fibers OptiLite™ and Odyssey 30 Holmium Laser System™ (Cook Medical Devices) was performed. Fragments were extracted with NCircle^®^ Delta Wire^®^ Nitinol Tipless Stone Extractor device (2.4F and 115 cm long-Cook Medical Devices). The complete absence of residual stones was confirmed by endoscopic vision and fluoroscopy.

**FIG. 3.**
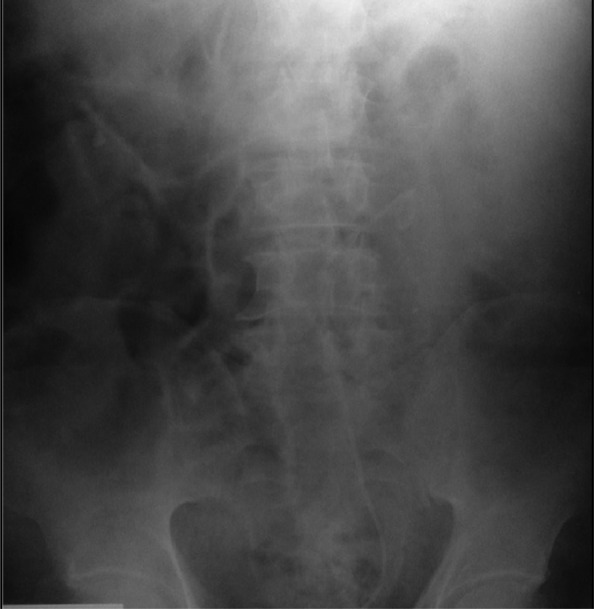
Postsurgical X-ray.

The procedure was completed with the placement of Double-J ureteral catheter 6F-26 cm (Cook Medical Devices) (Fig. 3).

A week after the procedure, the Double-J stent was removed without complications.

Considering the size and location of the stone, extracorporeal shockwave lithotripsy could have been the first line of treatment, but because of the size of the spleen, it was contraindicated.

Furthermore, percutaneous nephrolithotomy (PCNL) was also contraindicated, also due to the possibility of splenic injury. This information was provided by the CT scan, highlighting the importance of using higher quality imaging studies before treatment decision. The CT scan provides greater accuracy than ultrasonography and X-ray for the evaluation of the urinary tract,^1^ both in the preoperative stage for evaluation of access to be used and in the assessment of the distribution of solid and hollow neighboring viscera to avoid complications.

Due to the close anatomical relations that have kidney with neighboring organs, such as spleen, colon, liver, duodenum, and pleura, all these structures are likely to be injured during percutaneous puncture. Splenic injury is one of the major complications of percutaneous surgery. Fortunately, it is a rare complication. Treatment in this case ranges from expectancy to splenectomy.^2^ It is essential to reach an early diagnosis to prevent further complications. If hemodynamic instability occurs during the postoperative course of patients with left PCNL, it should be suspected splenic injury, even if patients presented with mild haematuria.^2,3^

Regardless the spleen size, punctures made in the 10th or 11th intercostal space carry the risk of splenic injury in up to 33% of cases.^2^

In recent decades, technologic advances have allowed the development of flexible instruments and laser devices for stone fragmentation. Over the years, devices have an increasingly smaller caliber and greater flexibility. RIRS has shown excellent results, generating a strong interest in urologists.^4^

RIRS offer fewer complications than percutaneous surgery, with a high rate of stone-free patients.^4^ Proper use of the different options available increases success and reduces complications of procedures.^4^

## Outcome

Performing preoperative CT allowed to discover the presence of unsuspected splenomegaly, which changes treatment tactics applied in this patient. RIRS allowed a stone-free patient without complications. Currently, the patient has no stone recurrence.

## Abbreviations Used

CT: computed tomography
PCNL: percutaneous nephrolithotomy
RIRS: retrograde intrarenal surgery
